# On Learning to Anticipate in Youth Sport

**DOI:** 10.1007/s40279-022-01694-z

**Published:** 2022-05-27

**Authors:** Tim Buszard

**Affiliations:** 1 Institute for Health and Sport, Victoria University, Melbourne, VIC Australia; 2 Game Insight Group, Tennis Australia, Melbourne, Australia

## Abstract

Elite sport offers a suitable setting to understand the ability to anticipate future events—a phenomenon that is central to animal life. Critically, however, whilst anticipation in sport has been studied for several decades, there have been few attempts to understand its development throughout childhood and adolescence. Additionally, whilst it is widely acknowledged that the need to anticipate emerges from temporal pressure, there has been no effort to understand the nonlinear effect that temporal demands have on the development of anticipatory skill. This is important as its consequences have different implications for sports authorities compared to an individual player. To bridge the gap in our understanding, this article draws attention to the mathematical concepts of concavity and convexity to explain the nonlinear relationship between temporal demands and the development of anticipatory skill. This viewpoint has implications for the design of junior sport, including the modification of rules, which has gained worldwide interest in recent years.

**Supplementary Information:**

The online version contains supplementary material available at 10.1007/s40279-022-01694-z.

## Key Points

**Table:** 

Elite athletes have a remarkable ability to anticipate future events, but our knowledge of when this skill develops across childhood and adolescence is limited.
I argue that the ability to anticipate develops in a nonlinear manner as a function of temporal pressure.
Temporal pressure can cause a system to be fragile or antifragile (with respect to developing anticipation skill), which has different implications for sports authorities compared to an individual player.

## Introduction

The ability to anticipate future events is central to animal life—whether it be anticipating a predator’s attack or the speed of an approaching car when crossing the road. Acts of remarkable anticipation are perhaps most evident in the sporting arena. Roger Federer has less than 500 ms to return a Novak Djokovic serve on the grass courts of Wimbledon; yet, amazingly, he is consistently in the right position at the right time to intercept the ball. This act of extreme skill has been well documented across many tasks, and it is a testament to the ability to exploit advanced information (e.g., sources of information from the game situation or an opponent’s kinematics) to guide the coordination of movements.

The field of anticipation research has advanced substantially since the seminal work by Jones and Miles [1], who investigated the ability to predict the landing location of tennis serves. A primary focus of the field has been to understand the information that expert performers use to guide anticipation (i.e., what do they *perceive*) and the development of training methodologies via the application of technology (i.e., can it be *improved*). The field, however, has fallen short on at least two accounts. First, there has been a lack of attention on the development of anticipation throughout an athlete’s formative years. That is, research has been biased towards detailing the qualities of expert athletes at the expense of understanding development. While this is a logical methodological approach to understanding anticipation, research is now at a point where understanding development is necessary. Second, the field has generally neglected the most obvious ingredient that influences the learning of anticipation—*temporal pressure*. This is not to say it is not recognised; in fact, it is quite the opposite—many researchers (and coaches) will confirm that temporal pressure is integral to developing anticipatory skills. Critically, though, there have been no attempts to quantify the nonlinear relationship between temporal pressure and the learning of anticipation.

In this article I draw the link between *learning* to exploit information arising prior to an event, which I define as our function $S(x)$, and temporal pressure, $x$. It is an extension of previous suggestions implying a link; but more significantly I bring into the conversation the concepts of concavity and convexity, or fragility and antifragility—mathematical concepts that describe the nonlinearity of outcomes [2–4]. In doing so I highlight the possible consequences from the environments we create in junior sport in developing the skill of anticipation.

## A Summary of Anticipation Research

There have been a number of reviews of anticipation in sport [5–10], so I will not attempt to replicate these; but I will provide a short synopsis of the current state of knowledge. We can describe anticipation as follows: it is the ability to utilise information arising before an event (say, before the server makes contact with the ball in tennis) to accurately predict a subsequent event (e.g., the ball’s landing location). Critically, however, anticipation in sport is not solely a prediction exercise—it requires the coupling of perception and action [11]. Hence, in our serve-return example, successful anticipation for the returner involves being perceptually attuned to key sources of pre-ball-flight information, such as the postural kinematics of the server, and this information guides the coordination of movement with future information (e.g., the trajectory of the ball) so that the player is in a better position to strike the ball.

Key sources of information used to guide anticipation can be described under two categories—kinematic and contextual. The former dominated our thinking during the first few decades of anticipation research [e.g., 12–15]. These studies revealed several regularities amongst expert performers; namely, a greater sensitivity to certain postural cues at specific moments [e.g., 1, 16–19]. More recently researchers have directed their attention towards the importance of contextual information, such as probabilistic intel derived by game situations and opponent tendencies [e.g., 20–25]. The challenge now for scientists is to understand how kinematic and contextual information are exploited simultaneously [e.g., 26].

It must also be highlighted that not all information is helpful; or, in *Gibsonian* language, some information is *specifying*, meaning it reliably predicts a subsequent event, and other information is *non-specifying*, meaning it *can* predict a subsequent event but it is less reliable [27]. The journey towards becoming an expert can therefore be viewed as a process of becoming attuned to *specifying* information or making better use of *non-specifying* information [29, 29]. Issues arise, however, when an opponent is deceptive, meaning the most salient information is hidden, leading to inaccurate anticipating, or perhaps more truthfully, excellent deception [30–33]. Anticipation is also inhibited when probabilistic information is incongruent with the event. For instance, if a skilled handball goalkeeper becomes aware that an opponent has a bias in the direction of their throws when shooting for goal, the goalkeeper will likely miss picking up the critical information that suggests the ball will be thrown in the other direction [21].

To conclude the short synopsis of anticipation research, a major quest stemming from this work has been the application of technology to enhance an athlete’s anticipatory skills. The typical training program has adopted a temporal occlusion paradigm, whereby the performer’s vision is occluded at specific moments, usually just prior to an event (e.g., immediately prior to the tennis player striking the ball). Initially the training involved the projection of vision onto a large screen, and players mimicked an action to indicate their anticipatory decision [for reviews, see 34–36]. There is some evidence, albeit weak, to suggest that the improvements observed in this training do transfer to the *real world* [19, 37–41] or to improved performance under pressure [42]. More recently research has directed its attention towards the use of technology to maintain perception–action coupling, whether it be via virtual-reality [43] or the use of occlusion goggles during real-world training [44]. The jury is still out as to whether these technologies improve an athlete’s anticipatory skill [45]. Consequently, as I argue next, our attention has been too heavily directed at finding *small gains*, rather than on the factor that I suspect has the largest impact on the development of anticipatory skill—*temporal pressure in youth sport.*

## When Do Players Learn to Anticipate in Sport?

Sports anticipation research has spanned over 50 years, yet few studies have investigated youth performers [12, 20, 39, 46–50]. This highlights a critical feature of the field: we have been overly focused on identifying the characteristics of expert performers (which is important) and understanding whether anticipatory skills of elite athletes can be enhanced, so that we have neglected the time of life where largest growth occurs—childhood and adolescents. Indeed, in many sports the mid-to-late teenage years are considered essential for development of expertise. More specifically, it is considered important during these years to specialise in a sport via an investment in deliberate practice [51]. It is therefore evident that this is a period when training can be highly impactful.

Of the studies that have investigated youth performers, some have adopted a cross-sectional design while others examined learning. The cross-sectional studies revealed the following: (1) skilled junior badminton players (aged 10–19 years) were no better than their novice counterparts in predicting the serve location from earlier time-points, but performance did differ when certain advanced information sources were removed [12]. This suggested that the skilled players were using different information sources (information from the opponent’s arm) to predict serve direction compared to the novices (information from the opponent’s racket). (2) Skilled adult tennis players outperformed lesser skilled adults in predicting serve location when vision was occluded pre-ball-flight, whereas this skill level difference was weak to non-existent in players aged 8–18 years [49]. (3) Skilled 12-year-old tennis players were unable to detect a pattern in service direction based on the match situation—the first serve of every game was directed down the T—but skilled 15-year-old’s did [20]. (4) Skilled 15-year-old cricket players did not display an advantage (relative to lesser skilled players of the same age) in predicting the bowler’s subsequent delivery based on kinematic information; however, this superior ability was evident for skilled under 18-year-old (U18) players and skilled adults [46]. (5) Skilled soccer players aged 8–16 years could more accurately predict the direction of passes before passes were executed compared to lesser skilled players of the same age [47]. The skilled players were also superior in a novel test that required participants to assign probabilities of the likely next action by attackers in a match. (6) Volleyball players in the U13, U15 and U17 age groups predicted shot location reliably better than chance, whereas U9 and U11 players did not [50].^1^

Before we draw conclusions from these studies, we need to take heed of methodological limitations and differences between sports. Each study adopted a video-based temporal occlusion paradigm to measure anticipation, with participants responding to the video via touching a screen or pen and paper. It has been established that the results derived from tasks that de-couple perception and action underestimate the expertise effect [52, 53]. Hence, if anything, these studies likely underestimate the ability to anticipate. Differences between sports are also noteworthy. Tennis and cricket are both sports that evoke high temporal demands at the elite level, but in juniors these sports have been traditionally played under full-sized (adult) conditions, meaning temporal demands are much lower. Comparatively, the nature of soccer (particularly small-sided games), with attackers and defenders constantly applying pressure to each other, means that junior soccer is likely to evoke higher temporal demands than junior cricket and tennis (if these sports are played under full-sized conditions). There was also a notable difference between the volleyball and soccer experimental tasks, and those in the cricket and tennis studies. In the former two sports, the actions perceived (setting the ball in volleyball and dribbling or passing in soccer) were arguably simpler than those in the cricket and tennis studies (fast bowling and serving). It is therefore possible that anticipatory performance reflects task difficulty. With these caveats in mind, we can deduce the following from the six cross-sectional studies: children *can* anticipate, but its development is not necessarily a by-product of playing sport and becoming skilled.

Other studies investigating anticipatory skill in youth performers focused on the efficacy of video-based temporal occlusion training under various instructional methods [39, 48]. Both studies targeted intermediately skilled tennis players, but the results were mixed. In both studies the instructional groups improved anticipatory performance from pre- to post-test during on-court tests more than a control group. Critically, however, the instructional groups either did not outperform a placebo group who simply watched vision of tennis matches [54] or were not compared to a placebo group, which therefore limits the generalisability of the results [39]. Nonetheless, for the purpose of the current discussion, these results further support the previous conclusion that children *can* improve their ability to anticipate, albeit the most effective use of video is less clear. At this juncture I must emphasise that it is not my intention to discuss the potential role of instructions; rather, my aim is to bring attention to the role of temporal pressure (as discussed in the next section), and I argue that without temporal pressure there is no need for instruction, as the need to anticipate would be superfluous.

## Temporal Pressure and the Youth Sport Environment

Temporal pressure is necessary to elicit anticipatory behaviour [55]. Perhaps the most eloquent display of this was the investigation by Florian Loffing on the left-handed advantage in professional sport [56]. Left-handed athletes are over-represented in a number of interceptive sports, and this is thought to be because athletes are less attuned to the behaviour of left-handed performers given that there are fewer left-handers in the general population. Significantly, Loffing [56] showed that the over-representation of left-handers was only apparent in interceptive sports that evoked the greatest temporal pressure, such as cricket (bowlers), baseball (pitchers) and table tennis, but not tennis, badminton or squash. This implies that the left-handed advantage arises when an athlete can impart high temporal pressure on their opponent.

Given the importance of temporal pressure for anticipatory behaviour to emerge, it has been remarked that temporal demands in junior sport may be insufficient to evoke anticipatory behaviour [20, 46]. Taking tennis as an example, the temporal demands on a full-sized court will be considerably less for a 10-year-old compared to a Federer versus Djokovic battle. This is, of course, obvious—professionals serve the ball with significantly more speed. Consequently, the temporal demands of a professional match are approximately 170% faster than a 10-year old’s match [57]. Similar differences were noted in cricket, with temporal demands beings 188% faster for professionals than 10-year-olds when playing on a full-sized pitch. (*Note:* I am not insinuating that the aim of junior sport should be to replicate the temporal demands of professional sport, as I will articulate more precisely later; this is merely an exercise to illustrate the difference.) There is of course one notable difference between professional athletes and children: professionals can organise their movements much faster than children; hence, temporal demands need to be considered relative to a performer’s motor ability. Nonetheless, given that it was found that skilled junior tennis and cricket players did not display anticipatory behaviour at a young age [20, 46], it seems possible that young players can achieve success without needing to anticipate. This raises the question: *could junior sport be modified to elicit the development of anticipatory skills?*

Studies investigating the junior sport playing environment have grown considerably over the past decade [58, 59]. The results provide a compelling account that appropriate modifications to task constraints, such as field size or equipment size, can allow children to play sport in a manner that better resembles a professional match. This implies that anticipatory skill might also emerge in youth sport if the game is modified appropriately. For example, modifying tennis by means of using scaled rackets, lower compression balls, lower nets and/or smaller courts has manifested in a number of desirable changes, including more aggressive stroke-play (e.g., faster rallies, more winners without an increase in errors, more approaches to the net, and more shots struck inside the baseline) [60–62], fewer errors [63], superior serving [61, 64, 65], and improved coordination [66, 67]. Similar results were evident when cricket was modified, with a shorter pitch leading to perception–action coupling more akin to professional cricket (i.e., short-pitched deliveries were played off the back foot rather than the front foot) [68], more accurate bowling, which also leads to batters having more opportunities to strike the ball [69, 70], and bowling with a technique that is deemed at less risk of injury [71]. Altogether, the constraints imposed in the junior sport environment play an important role in the development of skill.

## Measuring Anticipatory Behaviour

**Fig. 1:**
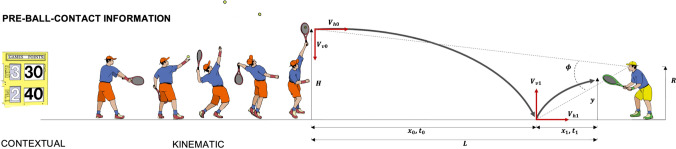
An illustration of the serve-return in tennis. A successful return requires the returner to coordinate their movements with the trajectory of the ball. Several sources of information are available from the ball based on the physics of ball flight, and this information describes *where* and *when* to strike the ball. If the initial velocity of the ball is high (relative to the returner’s action capabilities), the challenge for the returner is to perceive the ball’s trajectory as early as possible to allow sufficient time to coordinate their movements. The ability to exploit (reliable) pre-ball-flight information (kinematic or contextual) is therefore advantageous for performance insofar as it allows movements to be regulated with sufficient time to allow the skill to be executed successfully

Before discussing *the learning* of anticipatory skill, it is first important to consider how it is measured. Anticipation is inferred by the ability to exploit information arising before an event, such as the availability of ball flight information when returning a tennis serve. More precisely, successful anticipation is when a performer adapts their movements to time constraints based on the perception of kinematic or contextual information. The significance of adapting movements to time constraints must be acknowledged here. The reason we marvel at elite athletes is because the time required to perform a movement often exceeds the time window that the most salient information is available (see Fig. 1).

However, as a consequence of the focus on exploiting early-arising information, anticipation has often been assumed to be represented by an *earlier* initiation of movement in response to an event. In many cases, specifically those that involve extreme temporal demands, this is likely to be true. Initiating movement earlier provides more time to coordinate and execute the desired action. Nevertheless, initiating movement earlier cannot always be considered advantageous, as a less risky strategy is one whereby the performer moves *as late as possible given their action capabilities* [30, 72]. In other words, given that anticipation accuracy improves with the benefit of later arising perceptual information, it would be wise for a performer to use as much of this information as possible [73]. But, of course, there is a limit to this strategy; if one leaves it too late, they will not be able to coordinate their movements in sufficient time to execute the action (i.e., their action capabilities) [74, 75].

## Second-Order Effects: Convex Responses

**Fig. 2:**
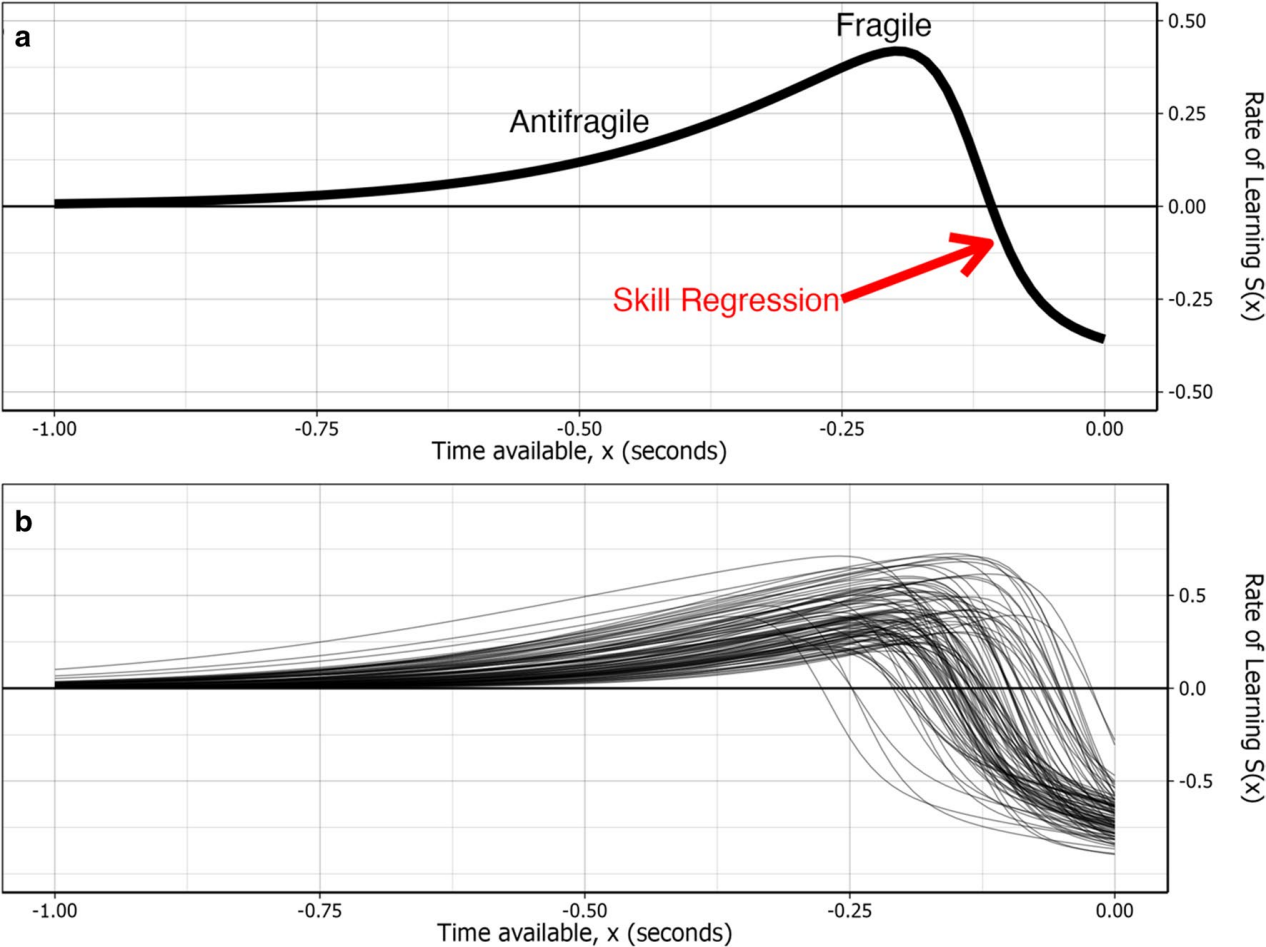
An illustration of the proposed relationship between rate of learning $S\left(x\right)$ and the time available to perform an action $\left(x\right).$ $S\left(x\right)$ is a function that describes the second-order consequences from temporal pressure $\left(x\right)$. Essentially, the function describes *rate of learning*, or more specifically the *rate of behavioural change* following exposure to $x$ (see footnote 3 for an outline of how this relates to the timescales of motor learning model). $S(x)$ is represented by the multiplication of two sigmoid functions, with $x$ describing the time between an event and action (i.e., the temporal pressure inflicted on the performer). Temporal pressure (i.e., the time available) can be measured via the spatiotemporal constraints of the task; namely distance (e.g., the length of the tennis court) and speed (e.g., the opponent’s service speed). It is important to recognise that distance is often (but not always) a stable variable (e.g., in tennis the serving distance will remain the same for the match based on court length) whereas speed is a stochastic variable. This has important implications for the design of junior sport (see Sect. 7). **a** Antifragility is defined by when the curve of $S\left(x\right)$ is $\ge 0$ and convex, whereas fragility is defined by when the curve of $S\left(x\right)$ is concave. $S\left(x\right)$ is also convex when < 0, but this cannot be considered *antifragile* since it represents skill regression. **b** $S\left(x\right)$ will differ for every individual as per the individual nature of motor learning curves

Nassim Taleb introduced the concepts of fragility (concavity) and antifragility (convexity) [2–4] (see Fig. 2). Derived from finance, they have since been applied to biological systems to describe the nonlinearity of consequences in response to stress [76]. A fragile system is one that exhibits more harm than benefits in response to stress whereas an antifragile system is the opposite; the benefits from stress outweigh the harm. The most critical aspect of these definitions is that there is asymmetry in the consequences. Importantly these concepts can be applied to many aspects of life, including our understanding of the link between temporal pressure and developing the ability to anticipate.

Biological systems are inherently antifragile. Taking strength training as an example, lifting a certain amount of weight can lead to greater benefit (muscle hypertrophy) than harm (muscle damage), but only up to a certain load. At this point the system becomes fragile, meaning there is greater risk of harm than benefit if the load increases by a small amount. These concepts are useful for understanding the nonlinearity of dose–response relationships. Applied to the development of anticipatory skill, for instance, they can help uncover whether a system is fragile or antifragile to a particular level of temporal stress.

Let us define the *learning* (or growth) of anticipatory skill as our function $S\left(x\right),$ where $x$ represents temporal pressure—the time available to perceive the most salient information (e.g., ball flight information when returning a tennis serve). The definition of our function is important: it represents the *rate of change* in the ability to initiate movement with sufficient time to accurately respond under high temporal constraints to allow an action to be coordinated as desired.^2^ To clarify further, high temporal constraints signify that the performer needs to exploit perceptual information arising before the most salient information becomes available. Additionally, it is important to recognise that this function describes a second-order effect of temporal pressure. It is not merely trial-to-trial behaviour; rather, it represents the rate of change over a longer timescale as a function of temporal pressure. This longer timescale can be viewed as the *slow learning curve* within the timescales of motor learning model [77, 78].^3^

The implication of this function is that there are situations whereby a player will (1) experience no change to anticipatory skill due to the temporal demands being insufficient, (2) benefit exponentially (with regards to learning anticipatory skill) when temporal pressure increases slightly—compared to when there is no variance in temporal pressure (i.e., change is convex); (3) benefit maximally at higher levels of temporal pressure, but this also comes with the risk of a substantial decrease in rate of learning if temporal pressure increases by a certain threshold (i.e., change is concave); and (4) experience a regression in skill when temporal pressure is too high.

Central to our understanding of fragility and antifragility is the conceptualisation of *risk.* The second situation above defines when the system is antifragile. This means that if time available ($x$) varies from trial-to-trial, the rate of learning $S\left(x\right)$ is more likely to benefit than experience harm. To explain further, let us assume the model in Fig. 2 is an accurate representation of a particular task, and for this task we aim to create a practice environment where temporal pressure equals 0.5 s (i.e., $x=-0.5$ in Fig. 2). Based on this $x$ value, we can then estimate rate of learning $S(x)$. However, quite often there will be some variation from trial-to-trial in temporal pressure (e.g., a tennis player will exhibit variations in serving speed across a series of serves), and *antifragility* occurs when a small increase in temporal pressure causes a greater (positive) response in $S(x)$ compared to a small decrease in temporal pressure. Indeed, this is what transpires at $x=-0.5$ in Fig. 2.

Comparatively, the third situation describes a fragile system: although the potential rate of learning is at its highest, there is also greater risk for a large reduction in the rate of learning if temporal pressure ($x$) varies by a certain threshold (meaning the time available is excessively short). Indeed, this situation can lead to a regression in skill (i.e., the fourth situation), which occurs when $S(x)$ < 0. It must be emphasised that whilst a fragile state can produce optimal outcomes, it can also produce considerably worse results under variable conditions. This, however, has greater implications for sports authorities designing the rules of junior sport compared to an individual player during a practice session (see next section).

The method to detect fragility and antifragility was proposed by Taleb [2, 4], and can be described mathematically as: if over a range $x\in \left[a,b\right],$ $\frac{1}{2}[(S\left(x+\Delta x\right)+\left(S\left(x-\Delta x\right)\right] \ge S\left(x\right),$ with ($x+\Delta x)$ and $\left(x-\Delta x\right)\in \left[a,b\right]$, then the system is classified as either fragile or antifragile depending on whether positive $S\left(x\right)$ values represent harm or benefit. In this article, positive values are considered beneficial since $S\left(x\right)>0$ represents learning and $S\left(x\right)<0$ represents skill regression.

The conceptualisation of learning as fragile or antifragile bears resemblance to the challenge-point hypothesis, which asserts that there exists a level of challenge where motor learning is optimised [80]. However, the (anti)fragility viewpoint is different for at least two reasons. First, if we define the *optimal* level of challenge as when learning is maximised, then the optimal level coincides with a fragile state (i.e., response is concave to stress)—a state that can (and should) be tested by an individual player, but not scaled to a large population of players (see next section). Second, whilst the challenge-point hypothesis provides a theoretical relationship between task difficulty and learning, (anti)fragility offers a robust mathematical link between probability and function. With this in mind, (anti)fragility offers a unique perspective for understanding perceptual-motor learning.

## Implications for Sport Authorities and the Player

Viewing the learning of anticipation via the concepts of fragility and antifragility has different implications for specific situations. I will make two contrasts—sports authorities and the individual player. The former considers $S\left(x\right)$ at the population level, whereas the latter focuses on the individual.

### Sports Authorities

The modification of junior sport rules has become popular at the level of governance over the past decade. By modification, I strictly mean the scaling of children’s sport so that the demands of the game better match children’s physical capabilities. In 2010, the International Tennis Federation (ITF) made the fifth rule change in the history of tennis, which mandated the use of lower compression balls in ITF-endorsed tournaments for players aged 10 years and younger. More recently, in 2018 Cricket Australia and the English Cricket Board changed the rules of junior cricket to enforce shorter pitches (amongst other rules). Decisions such as these have a widespread effect on children’s engagement in sport and the subsequent skills they develop. Indeed, there is arguably no other factor that can have such a profound positive impact on children’s sport than the scaling of equipment and play area [81]. Nonetheless, extra caution must be taken when deciding on these modifications, particularly when we consider fragility and antifragility.

**Fig. 3:**
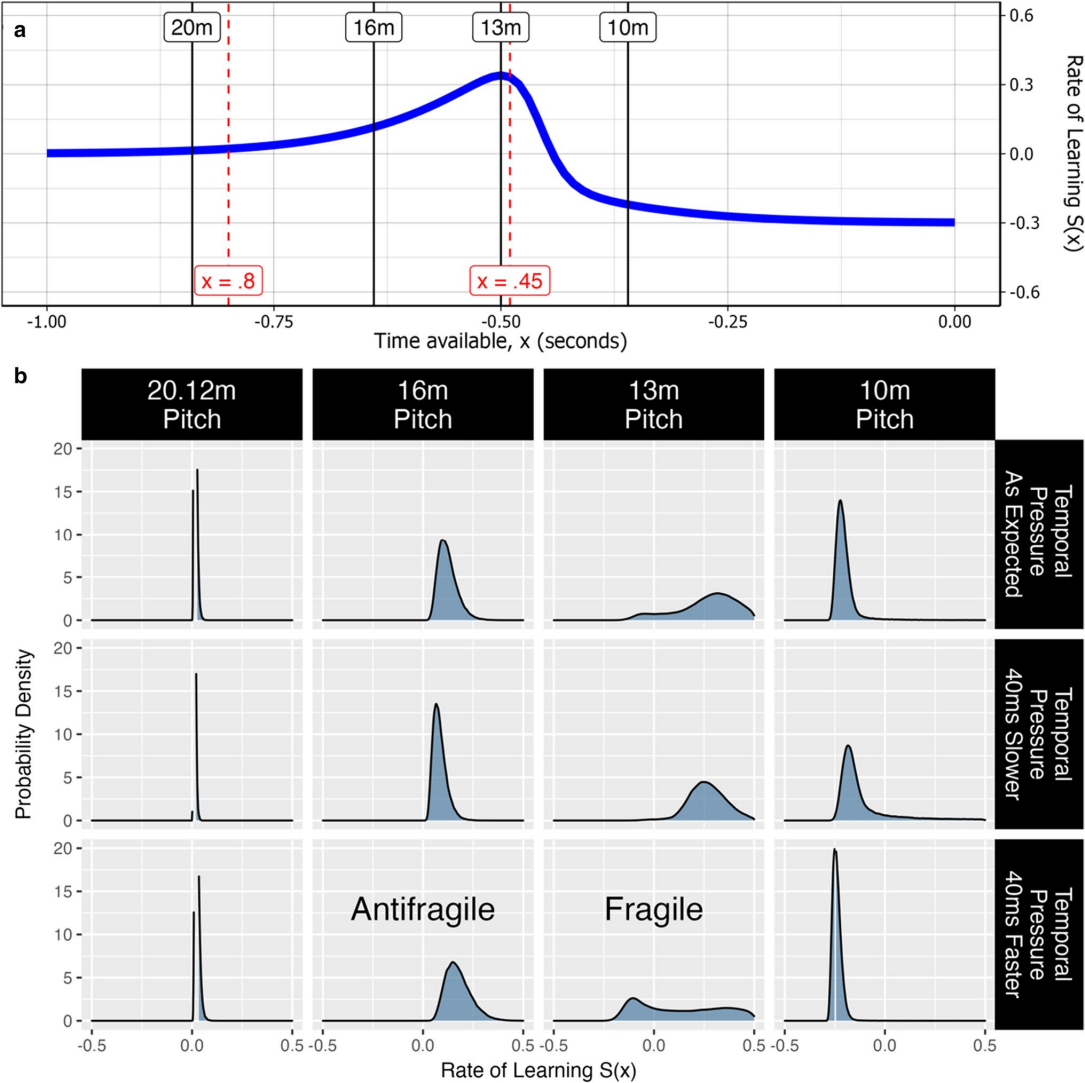
A junior cricket example to demonstrate how fragility and antifragility can be detected using methods developed by Taleb [2, 4]. **a** Estimated learning rate $S\left(x\right)$ was simulated for under 10-year-old (U11) cricket players when playing on various cricket pitches, with $S\left(x\right)$ = ($\frac{1}{1+{e}^{-11x-5}})(1-\frac{1.3}{1+{e}^{-65x-30}})$. The red dashed lines for $x$ = 0.8 and $x$ = 0.45 represent two of the assumptions that $S\left(x\right)$ was based on (see Sect. 7.1). **b** The probability density function of $S\left(x\right)$ was calculated when aiming to achieve specific temporal demands (i.e., time available, which represents ball flight duration). Ball flight duration was calculated based on previously reported parameters for U11 fast bowlers [69]: mean speed = 76 kph; mean release height = 163 cm. Ball flight distance represented pitch length minus 2.44 m (i.e., the distance between the popping crease and bowling crease at both ends). The headers along the top represent different pitch lengths, whereas the headers along the right represent the value for $x$. “40 ms slower” and “40 ms faster” represent a small change in $x$ from the expected value. The 13-m pitch is considered *fragile* since there is greater harm than benefit when $x$ varies, whereas the opposite is true for the 16-m pitch; hence this is *antifragile*. Note that the curve is convex for the 16-m pitch, whereas the curve is concave for the 13-m pitch. The curve is also convex for the 10-m pitch, but this also coincides with skill regression ($S\left(x\right)$< 0), which means it cannot be considered an antifragile state. See Online Supplementary Material for R code

To illustrate this further, if a governing body aims to *optimize* the dimensions of junior sport to maximise the development of anticipatory skill, they will likely place junior sport in a fragile state. This is simply due to the stochastic nature of $x$ (the variable describing speed). In other words, it is conceivable that $x$ will fluctuate more than the threshold governing the acceleration of harm due to the inherent variability that exists in children’s sport. This is not to say that sports organisations should not modify junior sport if the goal is to develop anticipatory skill. Figure 3 shows that making no change is likely to have minimal effect, whereas a certain level of modification can increase the probability of growth under variable conditions (*antifragility*).

The example in Fig. 3 simulates the estimated learning rate $S\left(x\right)$ for U11 cricket players when batting on various cricket pitch lengths. Estimated learning rate was based on the following assumptions [57]: (1) when temporal demands are low, there is little incentive to perceive the ball earlier and, consequently, the *potential for improving* anticipatory skill will be low; (2) the *potential for improving* anticipatory skill will remain low until temporal demands eclipse the total time required to perform the action (~ 600 ms for junior batters [82]) and the visuomotor delay (~ 200 ms based on adult batters [83]); (3) when temporal demands exceed the professional game (~ 450 ms), the *potential for improving* anticipatory skill in U11 players will decrease substantially as the ball will arrive too quickly. This situation represents when skill regression can occur. These assumptions led to $S\left(x\right)$ being defined by the curve as shown in Fig. 3a.

The mathematical method for detecting fragility and antifragility—as outlined in the previous section—was applied to calculate the probability density of $S\left(x\right)$ when aiming to achieve specific temporal demands ($x$) for various pitch lengths (Fig. 3b). Importantly, probability density was calculated for three situations within each pitch length: (1) temporal pressure occurs as expected, (2) temporal pressure is slower than expected (40 ms slower), and (3) temporal pressure is faster than expected (40 ms faster). The latter two scenarios represent small fluctuations in $x$ (i.e., $x\pm \Delta x$). Indeed, a change in $x$ by 40 ms can be caused by a 3- to 10-km change in bowling speed (depending on pitch length)—a practically conceivable amount.

Based on the assumptions for $S\left(x\right)$ and the typical bowling parameters for U11 fast bowlers [69], we might conclude that the *optimal* pitch length for improving anticipatory skill is approximately 13 m (see Fig. 3a). This condition is fragile, however, as there is greater risk of poorer outcomes if there is a small change to $x$ (e.g., if bowling speeds increase during a subsequent season of cricket for that age group). In other words, a small *decrease* in temporal pressure is more beneficial for $S\left(x\right)$ than a small *increase* in temporal pressure on a 13-m pitch. Indeed, increasing temporal pressure on the 13-m pitch heightens the probability of skill regression occurring. Comparatively, the 16-m pitch is an *antifragile* state as a small *increase* in temporal pressure is more beneficial for $S\left(x\right)$ than a small *decrease* in temporal pressure (see Fig. 3b). This therefore makes this pitch length desirable if creating rules for a large population of players (if the goal is to develop anticipatory skill).

### The Player

The story is different for the individual player. They can (and should) be far more aggressive since risk is bounded. Bounded risk simply means that the risk of skill regression is restricted to only one player, and almost always this player (or their coach) has the choice of manipulating temporal pressure if performance and learning are suffering (e.g., by increasing space during practice). Hence, the risk of skill regression can be easily mitigated. This contrasts with a large population of players who are playing under set rules and cannot easily regulate temporal pressure.

By aggressively exploring the boundaries of temporal pressure, the player can learn the boundaries of their action capabilities. More precisely, they can learn to calibrate their action system (i.e., time *required* to complete the task) with the situation (i.e., time *available* to complete the task), therein allowing more accurate perception between possible and impossible action. Given that the action system and the situation change over a longer time scale, it becomes important for the player to continually tinker with temporal pressure to recalibrate [84]. Tinkering in this sense can be viewed as constraint manipulation, such as reducing or increasing space.

Exploring the boundaries of temporal pressure can also be viewed through the lens of adaptive learning, whereby the aim is to continually adapt practice to maintain an appropriate level of challenge. From a dynamical systems viewpoint, this process has been described as *self-organised criticality*—practising at a difficulty level that ensures success is not 100% guaranteed (i.e., too easy), nor never achieved (i.e., too hard) [85, 86]. Somewhere in the middle is a *bi-stability* state*,* which means the performer exhibits both successful and unsuccessful solutions to the task (modelled as a saddle-node bifurcation) [87]. Importantly, with practice, the performer should learn to stabilise the successful solution in a more difficult setting than they previously could. By way of example, participants who self-regulated the difficulty of practice so that performance remained in a bi-stability state experienced superior motor learning compared to participants who were exposed to a prescribed practice plan of increasing difficulty [85]. The ability to self-control practice allowed participants to reduce task difficulty if it was too hard and vice versa if it was too easy, whereas this was not an option for the prescribed practice group. The results of two unique participants were also noteworthy. One participant never increased task difficulty in practice and, subsequently, learning was non-existent. Another participant demonstrated the opposite behaviour: they continually practiced at an excessively high difficulty level, meaning success was rarely achieved. This participant displayed negative learning—the equivalent of skill regression in this article.

## Conclusion

The purpose of this article was twofold. First, I wanted to shine a light on the field’s neglect to study the development of anticipation during childhood and adolescence. As a consequence, the link between temporal pressure in youth sport and the development of anticipatory skill is rarely discussed let alone quantified. Second, I believe the concepts of fragility and antifragility will improve our understanding of the development of anticipation (and, more broadly, skill acquisition). The most important implications depend on whether temporal pressure is being manipulated by a sports authority or an individual player. The former should be extra cautious when prescribing rules for the size of junior sport (whilst also realising the impact of full-sized conditions), whereas the latter should be far more aggressive given that risk is bounded. It is my belief that young performers can learn to anticipate, but *only if* the environment demands this behaviour.

## Supplementary Information

Below is the link to the electronic supplementary material.

Supplementary file1 (R 14 KB)

## Footnotes

1 De Waelle et al. [50] reported that 17% of U11 players and 36% of U13 players accurately predicted the opponent’s passing option better than chance, therein implying that some children in these age groups had acquired the ability to anticipate under high temporal constraints. However, a simulation of the task (40 trials with four possible responses) revealed that a random response strategy generated an overall score that was better than chance (25%) in 42% of attempts (SD = 1%) (see Online Supplementary Material for R code of the simulation). Hence, we cannot conclude that the U9 and U11 players in this study had developed the ability to anticipate. Comparatively, 70% of U13 performed better than chance, so we can safely conclude that these players possessed the ability to anticipate in this task.

2 Initiating movement with sufficient time to accurately respond is captured by the calibrating variable within the affordance-based control model of anticipatory skill. Van der Kamp et al. [74] proposed a differential equation to explain the required velocity by a goalkeeper to save a penalty kick: ${V}_{\mathrm{GK}}^{*}=c(\frac{{x}_{\mathrm{B}}- {x}_{\mathrm{GK}}}{{t}_{\mathrm{B}}+ {t}_{\mathrm{GK}}})$. ${V}_{\mathrm{GK}}^{*}$ represents the required lateral velocity to save the ball, as governed by the distance to cover ${(x}_{\mathrm{B}}- {x}_{\mathrm{GK}})$ in a given amount of time ${(t}_{\mathrm{B}}+ {t}_{\mathrm{GK}})$. Importantly, $c$ represents a calibrating variable that scales the required velocity with the performer’s maximum capabilities. Although this equation was developed for goalkeeping, it can easily be translated to other tasks.

3 The motor learning model of multiple timescales asserts that performance changes along distinctly different timescales, such as *fast adaptation* and *slow learning* [78, 79]*.* Fast adaptation refers to transient changes in performance that occur during initial practice repetitions (often referred to as warm-up decrement), whereas slow learning refers to permanent changes that occur across days, weeks and years. Solutions for each timescale are combined in the model by applying the principle of superposition. An example of the slow learning timescale is: $V\left(n\right)= {V}_{\mathrm{Inf}}+a{e}^{-\gamma n}. V\left(n\right)$ refers to the performance outcome variable after $n$ trials; ${V}_{\mathrm{Inf}}$ is the asymptotic target value that $V\left(n\right)$ converges towards; $a$ is the initial performance value; and the exponent $\gamma$ describes rate of change. Hence, rate of change captured by $S\left(x\right)$ in Fig. 2 can be viewed as the slow learning exponent ($\gamma$).

## Declarations

### Availability of data and material

No data were collected for this article.

### Code availability

R code is provided as Online Supplementary Material.

## References

[1] Jones C, Miles T (1978). Use of advance cues in predicting the flight of a lawn tennis ball. J Hum Mov Stud.

[2] Taleb NN (2012). Antifragile: how to live in a world we don't understand.

[3] Taleb NN. (Anti) fragility and convex respones in medicine. In: International Conference on Complex Systems. Springer; 2018. p. 299–325.

[4] Taleb NN, Douady R (2013). Mathematical definition, mapping, and detection of (anti)fragility. Quantitative Finance.

[5] Loffing F, Cañal-Bruland R (2017). Anticipation in sport. Curr Opin Psychol.

[6] Williams AM, Jackson RC (2019). Anticipation in sport: fifty years on, what have we learned and what research still needs to be undertaken?. Psychol Sport Exerc.

[7] Abernethy B, Farrow D, Mann DL, Erickson K, Hoffman RR, Kozbelt A, Williams AM (2018). Superior anticipation. Cambridge handbooks in psychology. The Cambridge handbook of expertise and expert performance.

[8] Mann DL, Savelsbergh GJ, Baker J, Farrow D (2015). Issues in the measurement of anticipation. Routledge international handbooks Routledge handbook of sport expertise.

[9] Cañal-Bruland R, Mann DL (2015). Time to broaden the scope of research on anticipatory behavior: a case for the role of probabilistic information. Front Psychol..

[10] Müller S, Abernethy B (2012). Expert anticipatory skill in striking sports: a review and a model. Res Q Exerc Sport.

[11] Van der Kamp J, Rivas F, Van Doorn H, Savelsbergh G (2008). Ventral and dorsal system contributions to visual anticipation in fast ball sports. Int J Sport Psychol.

[12] Abernethy B (1988). The effects of age and expertise upon perceptual skill development in a racquet sport. Res Q Exerc Sport.

[13] Abernethy B (1990). Anticipation in squash: differences in advance cue utilization between expert and novice players. J Sports Sci.

[14] Abernethy B, Russell DG (1987). Expert-novice differences in an applied selective attention task. J Sport Exerc Psychol.

[15] Abernethy B, Russell DG (1987). The relationship between expertise and visual search strategy in a racquet sport. Hum Mov Sci.

[16] Müller S, Abernethy B, Farrow D (2006). How do world-class cricket batsmen anticipate a bowler's intention?. Q J Exp Psychol.

[17] Huys R, Cañal-Bruland R, Hagemann N, Beek PJ, Smeeton NJ, Williams AM (2009). Global information pickup underpins anticipation of tennis shot direction. J Mot Behav.

[18] Abernethy B, Zawi K, Jackson RC (2008). Expertise and attunement to kinematic constraints. Perception.

[19] Williams AM, Ward P, Knowles JM, Smeeton NJ (2002). Anticipation skill in a real-world task: measurement, training, and transfer in tennis. J Exp Psychol Appl.

[20] Farrow D, Reid M (2012). The contribution of situational probability information to anticipatory skill. J Sci Med Sport.

[21] Mann DL, Schaefers T, Cañal-Bruland R (2014). Action preferences and the anticipation of action outcomes. Acta Physiol (Oxf).

[22] Milazzo N, Farrow D, Ruffault A, Fournier JF (2016). Do karate fighters use situational probability information to improve decision-making performance during on-mat tasks?. J Sports Sci.

[23] Gray R (2002). Behavior of college baseball players in a virtual batting task. J Exp Psychol Hum Percept Perform.

[24] Loffing F, Sölter F, Hagemann N, Strauss B (2016). On-court position and handedness in visual anticipation of stroke direction in tennis. Psychol Sport Exerc.

[25] Loffing F, Hagemann N (2014). On-court position influences skilled tennis players’ anticipation of shot outcome. J Sport Exerc Psychol.

[26] Helm F, Cañal-Bruland R, Mann DL, Troje NF, Munzert J (2020). Integrating situational probability and kinematic information when anticipating disguised movements. Psychol Sport Exerc.

[27] Gibson JJ (1966). The senses considered as perceptual systems.

[28] van der Kamp J, Renshaw I, Baker J, Farrow D (2015). Information-movement coupling as a hallmark of sport expertise. Routledge handbook of sport expertise.

[29] Van der Kamp J, Oudejans R, Savelsbergh G (2003). The development and learning of the visual control of movement: an ecological perspective. Infant Behav Dev.

[30] Dicks M, Davids K, Button C (2010). Individual differences in the visual control of intercepting a penalty kick in association football. Hum Mov Sci.

[31] Dicks M, Button C, Davids K (2010). Availability of advance visual information constrains association-football goalkeeping performance during penalty kicks. Perception.

[32] Bishop DT, Wright MJ, Jackson RC, Abernethy B (2013). Neural bases for anticipation skill in soccer: an fMRI study. J Sport Exerc Psychol.

[33] Jackson RC, Warren S, Abernethy B (2006). Anticipation skill and susceptibility to deceptive movement. Acta Physiol (Oxf).

[34] Hadlow SM, Panchuk D, Mann DL, Portus MR, Abernethy B (2018). Modified perceptual training in sport: a new classification framework. J Sci Med Sport..

[35] Broadbent DP, Causer J, Williams AM, Ford PR (2015). Perceptual-cognitive skill training and its transfer to expert performance in the field: future research directions. Eur J Sport Sci.

[36] Dicks M, van der Kamp J, Withagen R, Koedijker J (2015). Can we hasten expertise by video simulations? Considerations from an ecological psychology perspective. Int J Sport Psychol..

[37] Brenton J, Müller S, Harbaugh AG (2019). Visual-perceptual training with motor practice of the observed movement pattern improves anticipation in emerging expert cricket batsmen. J Sports Sci.

[38] Gabbett T, Rubinoff M, Thorburn L, Farrow D (2007). Testing and training anticipation skills in softball fielders..

[39] Smeeton NJ, Williams AM, Hodges NJ, Ward P (2005). The relative effectiveness of various instructional approaches in developing anticipation skill. J Exp Psychol Appl.

[40] Müller S, Gurisik Y, Hecimovich M, Harbaugh AG, Vallence A-M (2019). Individual differences in short-term anticipation training for high-speed interceptive skill. J Motor Learn Dev.

[41] Hopwood MJ, Mann DL, Farrow D, Nielsen T (2011). Does visual-perceptual training augment the fielding performance of skilled cricketers?. Int J Sports Sci Coach.

[42] Abernethy B, Schorer J, Jackson RC, Hagemann N (2012). Perceptual training methods compared: the relative efficacy of different approaches to enhancing sport-specific anticipation. J Exp Psychol Appl.

[43] Gray R (2017). Transfer of training from virtual to real baseball batting. Front Psychol..

[44] Mann DL, Abernethy B, Farrow D, Davis M, Spratford W (2010). An event-related visual occlusion method for examining anticipatory skill in natural interceptive tasks. Behav Res Methods.

[45] Renshaw I, Davids K, Araújo D, Lucas A, Roberts WM, Newcombe DJ (2019). Evaluating weaknesses of “Perceptual-Cognitive Training” and “Brain Training” methods in sport: an ecological dynamics critique. Front Psychol.

[46] Weissensteiner J, Abernethy B, Farrow D, Müller S (2008). The development of anticipation: a cross-sectional examination of the practice experiences contributing to skill in cricket batting. J Sport Exerc Psychol.

[47] Ward P, Williams AM (2003). Perceptual and cognitive skill development in soccer: the multidimensional nature of expert performance. J Sport Exerc Psychol.

[48] Farrow D, Abernethy B (2002). Can anticipatory skills be learned through implicit video based perceptual training?. J Sports Sci.

[49] Tenenbaum G, Sar-El T, Bar-Eli M (2000). Anticipation of ball location in low and high-skill performers: a developmental perspective. Psychol Sport Exerc.

[50] De Waelle S, Warlop G, Lenoir M, Bennett SJ, Deconinck FJ (2021). The development of perceptual-cognitive skills in youth volleyball players. J Sports Sci..

[51] Helsen WF, Starkes JL, Hodges NJ (1998). Team sports and the theory of deliberate practice. J Sport Exerc Psychol.

[52] Mann DTY, Williams AM, Ward P, Janelle CM (2007). Perceptual-cognitive expertise in sport: a meta-analysis. J Sport Exerc Psychol.

[53] Travassos B, Araújo D, Davids K, O'Hara K, Leitão J, Cortinhas A (2013). Expertise effects on decision-making in sport are constrained by requisite response behaviours—a meta-analysis. Psychol Sport Exerc.

[54] Jackson R (2003). Evaluating the evidence for implicit perceptual learning: a re-analysis of Farrow and Abernethy (2002). J Sports Sci.

[55] Triolet C, Benguigui N, Le Runigo C, Williams AM (2013). Quantifying the nature of anticipation in professional tennis. J Sports Sci.

[56] Loffing F (2017). Left-handedness and time pressure in elite interactive ball games. Biol Let.

[57] Buszard T, Whiteside D, Krause L, Reid M (2011). Formalising a hypothesis for the development of anticipatory skill in time-constrained interceptive actions: Implications for the design of junior sport. Psychol Sport Exerc..

[58] Buszard T, Farrow D, Reid M (2020). Designing junior sport to maximize potential: the knowns, unknowns, and paradoxes of scaling sport. Front Psychol..

[59] Buszard T, Reid M, Masters RSW, Farrow D (2016). Scaling the equipment and play area in children’s sport to improve motor skill acquisition: a systematic review. Sports Med.

[60] Timmerman E, De Water J, Kachel K, Reid M, Farrow D, Savelsbergh G (2015). The effect of equipment scaling on children’s sport performance: the case for tennis. J Sports Sci.

[61] Limpens V, Buszard T, Shoemaker E, Savelsbergh GJP, Reid M (2018). Scaling constraints in junior tennis: the influence of net height on skilled players’ match-play performance. Res Q Exerc Sport.

[62] Kachel K, Buszard T, Reid M (2015). The effect of ball compression on the match-play characteristics of elite junior tennis players. J Sports Sci.

[63] Fitzpatrick A, Davids K, Stone JA (2017). Effects of Lawn Tennis Association mini tennis as task constraints on children’s match-play characteristics. J Sports Sci.

[64] Gimenez-Egido JM, Ortega-Toro E, Palao JM, Torres-Luque G (2020). Effect of scaling equipment on U-10 players tennis serve during match-play: a nonlinear pedagogical approach. Chaos Solitons Fractals.

[65] Gimenez-Egido JM, Ortega-Toro E, Palao JM, Verdú-Conesa I, Torres-Luque G (2020). Effect of modification rules in competition on technical-tactical action in young tennis players (under-10). Front Psychol.

[66] Buszard T, Garofolini A, Reid M, Farrow D, Oppici L, Whiteside D (2020). Scaling sports equipment for children promotes functional movement variability. Sci Rep.

[67] Buszard T, Garofolini A, Whiteside D, Farrow D, Reid M (2010). Children’s coordination of the “sweet spot” when striking a forehand is shaped by the equipment used. Sci Rep..

[68] Harwood MJ, Yeadon MR, King MA (2019). A shorter cricket pitch improves decision-making by junior batters. J Sports Sci.

[69] Harwood MJ, Yeadon MR, King MA (2018). Does shortening the pitch make junior cricketers bowl better?. J Sports Sci.

[70] Harwood MJ, Yeadon MR, King MA (2018). Reducing the pitch length: Effects on junior cricket. Int J Sports Sci Coach.

[71] Elliott B, Plunkett D, Alderson J (2005). The effect of altered pitch length on performance and technique in junior fast bowlers. J Sports Sci.

[72] Wilson R, Dicks M, Milligan G, Poolton J, Alder D (2018). An examination of action capabilities and movement time during a soccer anticipation task. Mov Sport Sci Sci Motricité.

[73] Navia JA, Dicks M, van der Kamp J, Ruiz LM (2017). Gaze control during interceptive actions with different spatiotemporal demands. J Exp Psychol Hum Percept Perform.

[74] Van Der Kamp J, Dicks M, Navia JA, Noël B (2018). Goalkeeping in the soccer penalty kick. German J Exerc Sport Res.

[75] Zheng R, de Reus C, van der Kamp J (2021). Goalkeeping in the soccer penalty kick: the dive is coordinated to the kicker's non-kicking leg placement, irrespective of time constraints. Hum Mov Sci.

[76] Kiefer AW, Silva PL, Harrison HS, Araújo D (2018). Antifragility in sport: leveraging adversity to enhance performance.. Sport Exerc Perform Psychol.

[77] Newell KM, Liu Y-T, Mayer-Kress G (2001). Time scales in motor learning and development. Psychol Rev.

[78] Newell KM, Mayer-Kress G, Hong SL, Liu Y-T (2009). Adaptation and learning: characteristic time scales of performance dynamics. Hum Mov Sci..

[79] Verhoeven FM, Newell KM (2018). Unifying practice schedules in the timescales of motor learning and performance. Hum Mov Sci.

[80] Guadagnoli MA, Lee TD (2004). Challenge point: a framework for conceptualizing the effects of various practice conditions in motor learning. J Mot Behav.

[81] Reid M, Buszard T, Farrow D (2018). Learning, activity… and injury? Caring for young athletes through appropriately designed modified (developmental) sport. Br J Sports Med.

[82] Pinder RA, Renshaw I, Davids K (2009). Information–movement coupling in developing cricketers under changing ecological practice constraints. Hum Mov Sci..

[83] McLeod P (1987). Visual reaction time and high-speed ball games. Perception.

[84] Fajen BR (2008). Perceptual learning and the visual control of braking. Percept Psychophys..

[85] Liu Y-T, Luo Z-Y, Mayer-Kress G, Newell KM (2012). Self-organized criticality and learning a new coordination task. Hum Mov Sci.

[86] Newell KM, Liu Y-T, Mayer-Kress G (2009). Time scales, difficulty/skill duality, and the dynamics of motor learning.

[87] Liu Y-T, Mayer-Kress G, Newell KM (2010). Bi-stability of movement coordination as a function of skill level and task difficulty. J Exp Psychol Hum Percept Perform.

